# Evaluation of compartmentalization systems to study microbial interactions

**DOI:** 10.1038/s41598-025-32560-3

**Published:** 2025-12-18

**Authors:** Miguel Mejias-Ortiz, Ana Perea-Martínez, Ramon Gonzalez, Pilar Morales

**Affiliations:** Instituto de Ciencias de la Vid y del Vino (CSIC, Universidad de La Rioja, Gobierno de La Rioja), Logroño, La Rioja Spain

**Keywords:** Microbial interaction, Wine fermentation, Cell-to-cell contact, Compartmentalization devices, Yeast, Cross-flow filtration, Biological techniques, Biotechnology, Microbiology

## Abstract

With some exceptions, wine fermentation has increasingly relied on the use of *Saccharomyces cerevisiae* starters since the last decades of the past century. However, there is growing interest on the understanding of spontaneous wine fermentation, as well as on the use of complementary non-*Saccharomyces* wine starters. This in turn raises the question of the importance of interspecific interactions in winemaking and the underlying mechanisms. An important question about these interspecies recognition mechanisms is whether or not it is mediated by physical contact between yeast cells. To address this topic, different laboratories have developed diverse devices to cultivate at least two yeast species in the same growth medium without cell-to-cell contact between them. In this work, we compared four of the most popular systems and found that one of them (twin-bottles exchange through a flat membrane) showed very limited metabolite exchange. Among the other systems (Transwell, dialysis tube, or active exchange through hollow fiber cross-flow filtration devices), each one showed specific characteristics that made them more or less suitable, depending on the objectives of each experiment. The option showing the best versatility and efficiency was the use of active exchange. Our results highlight the importance of carefully characterizing the compartmentalization system when drawing conclusions about the impact of cell-to-cell contact in fermentation experiments.

**Supplementary Information:**

The online version contains supplementary material available at 10.1038/s41598-025-32560-3.

## Introduction

For many years, the conventional approach to wine biotechnology consisted of driving a naturally multi-species microbial community into a nearly pure culture using selected *Saccharomyces cerevisiae* starters^1^. However, the trend over the last twenty-five years is to include new yeast species as co-starters in order to recover the alleged “wild” character of traditional uninoculated wines, among other claims related to wine quality and technology^2^.

On the other hand, there is a growing interest in the study of microbial ecology during wine fermentation, fuelled by the power of metabarcoding and metagenomic analyses^3^. This responds both to a biotechnological interest in better understanding a process with important economic implications, and to the advantages of wine fermentation as a relatively simple, yet natural, model microbial ecosystem^4,5^.

In this context, the mechanisms involved in microbial interactions have also become the subject of increasing interest^6–8^. One issue that arises repeatedly when approaching the study of this topic is the need for physical contact to trigger the response. The answer to this question is relevant to guide further research on the interaction mechanisms. For example, it played a critical role in the identification of the involvement of *S. cerevisiae* GAPDH-derived peptides on interspecific interactions^9^.

To address the study of the effect of cell-to-cell contact, devices that allow separation of cells into different compartments while allowing free circulation of the medium are required. There are examples in the literature in which the mixed culture shows differential characteristics from membrane-separated cultures, while there are other examples in which no such differences are found^10,11^. The disparity in the reported results was to be expected, given the diversity of species, strains and growth or environmental conditions tested. However, some of them could be related to the choice of compartmentalization devices. In fact, when trying to develop a compartmentalization system for our studies, we realized that some of the published systems were not fit for purpose, particularly considering the metabolite exchange rate.

For this reason, we decided to perform a systematic comparison of compartmentalization systems that would be useful both for us and for other research groups interested in the subject of cell-to-cell contact and its implication in wine yeast interactions.

## Materials and methods

### Strains, culture media, and model solution

*S. cerevisiae* FX10 (Laffort, Bordeaux, France) was used as a representative commercial wine yeast starter. Synthetic must (MS300) recipe was based on Beltran et al.^12^ and Rossignol et al.^13^. It contained (per litre): sugars (glucose 100 g, fructose 100 g); acids (DL-malic acid 5 g, citric acid 0.5 g, tartaric acid 3 g); oligo-elements (KH_2_PO_4_ 0.75 g, K_2_SO_4_ 0.5 g, MgSO_4_·7H_2_O 0.25 g, CaCl_2_·2H_2_O 0.16 g, NaCl 0.2 g, MnSO_4_·H_2_O 4 mg, ZnSO_4_·7H_2_O 4 mg, CuSO_4_·5H_2_O 1 mg, KI 1 mg, CoCl_2_·6H_2_O 0.4 mg, H_3_BO_3_ 1 mg, (NH_4_)_6_Mo_7_O_24_ 1 mg); nitrogen sources for 300 YAN (120 mg from NH_4_Cl + 180 mg from amino acids): NH_4_Cl 0.46 g, Alanine (Ala) 145.6 mg, Arginine (Arg) 368.0 mg, Aspartic acid (Asp) 44.2 mg, Cysteine (Cys) 20.8 mg, Glutamine (Gln) 499.2 mg, Glutamic acid (Glu) 119.5 mg, Glycine (Gly) 18.2 mg, Histidine (His) 33.8 mg, Isoleucine (Ile) 32.5 mg, Leucine (Leu) 48.0 mg, Lysine (Lys) 16.9 mg, Methionine (Met) 31.2 mg, Phenylalanine (Phe) 37.7 mg, Proline (Pro) 599.3 mg, Serine (Ser) 78.0 mg, Threonine (Thr) 75.4 mg, Tryptophan (Trp) 175.0 mg, Tyrosine (Tyr) 19.5 mg, Valine (Val) 44.2 mg; vitamins (Myo-inositol 20 mg, Calcium Pantothenate 1.5 mg, Thiamine hydrochloride 250 µg, Nicotinic acid 2 mg, Pyridoxine hydrochloride 250 µg, Biotine 3 µg), anaerobiosis factors (ergosterol 15 mg, sodium oleate 4.85 µg, Tween 80 0.5 mL); pH adjusted at 3.3 with NaOH. Oligo-elements, vitamins, and amino acids were added from concentrated stock solutions in water, filter sterilized. Solution of anaerobiosis factors was prepared 1000X in ethanol, kept at 4 °C, and warmed to 70 °C just before addition. The medium was sterilized by filtration through a 0.2 µm Nalgene Rapid-flow filtration device (ThermoFisher Scientific, Dreieich, Germany)^14^.

Pre-cultures were grown in 50 mL centrifuge tubes with 20 mL of YPD (20 g/L glucose, 20 g/L peptone and 10 g/L yeast extract) at 25 °C for 48 h without shaking. Prior to inoculation in synthetic must, the precultures were centrifuged at 4500 × g for 5 min at 10 °C, followed by two washes with sterile distilled water.

The model solution for the evaluation of metabolite exchange rates contained (per litre): glucose 50 g, fructose 50 g, glycerol 10 g, acetic acid 2 g, and ethanol 200 mL. To rule out any leakage in the systems, yeast cells were also incorporated into this model solution to an OD_600_ between 2.5 and 4. The concentration of ethanol in the model solution prevents yeast growth.

### Compartmentalization devices

**Fig. 1:**
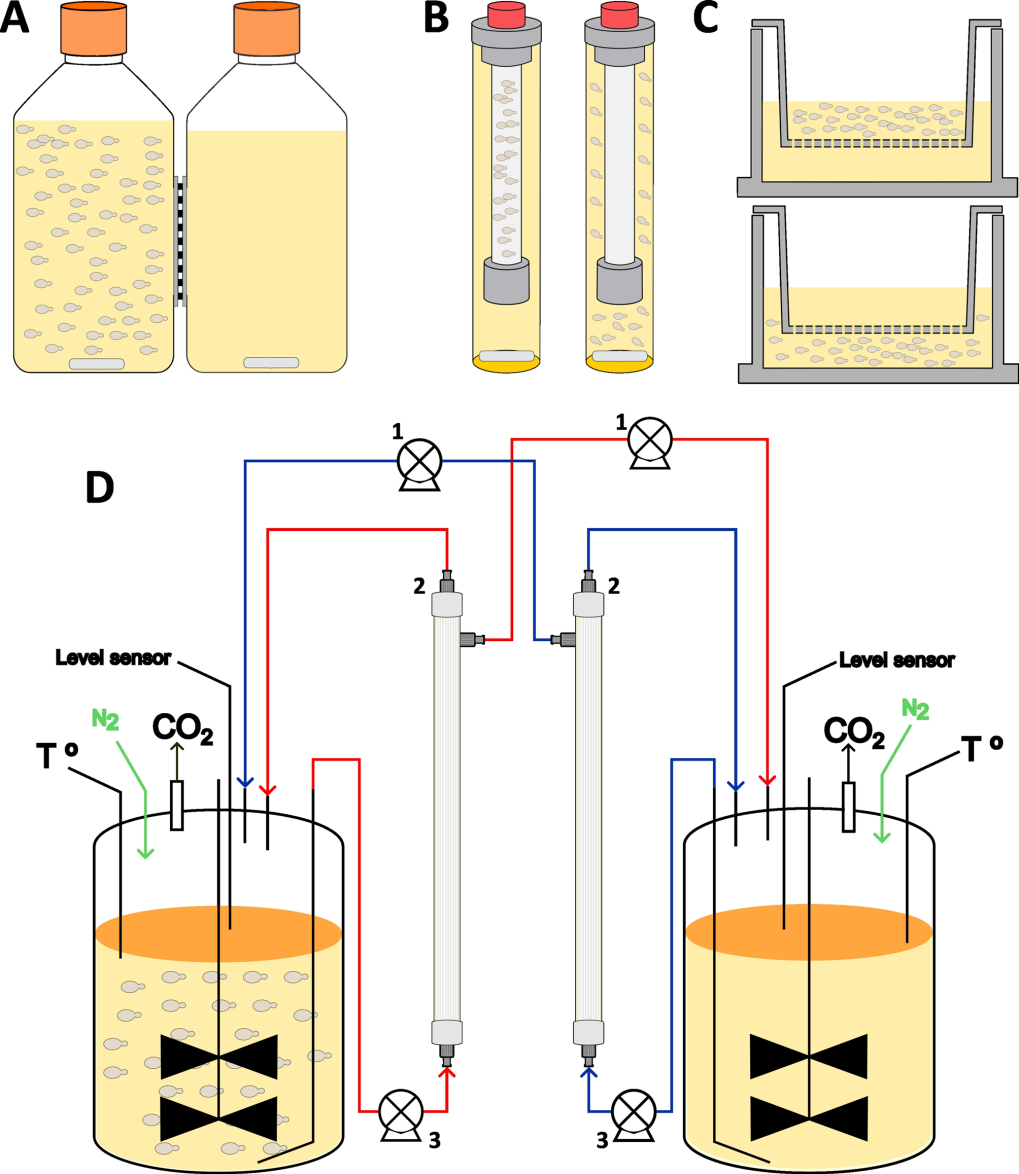
Schemes of the compartmentalization devices tested. (**A**): Twin-bottles system. (**B**): Dialysis system. Left scheme shows the inner compartment with cells and right scheme shows the outer compartment. These configurations correspond to the dialysis inside and dialysis outside tests. (**C**): Transwell system. The upper system corresponds to the Transwell inside test and the lower system corresponds to the Transwell outside test. (**D**): Bioreactors connected with cross-flow filtration (CFF) devices. 1: Integrated bioreactor peristaltic pump. 2: CFF device. 3: Watson-Marlow 120S peristaltic pump.

The four compartmentalization devices assayed in this work are shown schematically in Fig. 1. Whenever possible, the most similar pore size has been maintained between systems. A detailed description of each one is shown below.

Twin-bottles (Fig. 1A). This system consisted of two 250 mL polycarbonate bottles (ref. 431431 Corning, Somerville, MA) connected through a 32 mm orifice with a 47 mm filter with a pore size of 0.65 µm (ref. DAWP04700, Millipore, Burlington, MA). To ensure tightness, a 70 × 20 mm flat rubber gasket was placed on each bottle. In addition, a 36 × 3.5 mm O-ring was placed between the filter and the flat gasket of one of the bottles, and the whole system was tightened together with two zip ties. Prior to mount the device, the parts that conform the system were sanitized with 70% ethanol by submersion. Then, were let dry in a laminar flow cabinet. The membrane was autoclaved inside an aluminium foil envelope. The mounting was performed inside a laminar flow cabinet to ensure the system sterility. The flasks were filled with 250 mL of model solution or water and placed on magnetic stirrers to enhance metabolite exchange across the membrane.

Dialysis (Fig. 1B). The dialysis system used was the Float-a-Lyzer (ref. G235062, Repligen, Waltham, MA) with an internal volume of 5 mL and a pore size of 1000 kDa. The protective tube of the dialysis system was used as container for the 15 mL external solution. Magnetic stirring was used, with a magnet at the bottom of the tube. Prior to use, the membranes were prepared following the manufacturer’s user guide.

Transwell (Fig. 1C). A Transwell plate with 6 wells and a pore size of 0.4 µm was used (Costar ref. 3412 Corning, Somerville, MA). Each well was filled with 2.5 mL and its insert with 1.5 mL.

Bioreactors (Fig. 1D). Two 250 mL bioreactors (MiniBio, Applikon, Delft, Netherlands) were connected through two hollow fiber cross-flow filtration (CFF) systems. This type of filter allows for the filtering of great volumes of media whilst preventing the filter from clogging, making it appropriate for continuous filtration throughout fermentation. The cross-flow modules used were the MicroKros® hollow fiber filter module (ref. C02-E65U-07-N Repligen, Waltham, MA) with a cut-off size of 0.65 µm and a surface area of 15 cm^2^. Each system includes two peristaltic pumps. A pump (Watson-Marlow 120S, Marlow, UK) represented in Fig. 1D as “pump 3”, runs continuously at 50 rpm to push the media through the filtration system at a suitable flow rate. The second pump (“pump 1” in Fig. 1D) is part of the bioreactor control that receives the filtered media and responds to a level sensor (Fig. 1D). The speed and level sensors of these pumps were adjusted to keep the volume constant (200 mL) in each bioreactor.

### Metabolite exchange and fermentation experiments

Initial experiments consisted of filling one compartment with water and the other one with the model solution and running the system for 24 h at room temperature. No gas flow was used in the bioreactors for these experiments. For asymmetric systems (dialysis and Transwell), both possibilities were tested, i.e. model solution in the inner or outer compartment. Samples for metabolite quantification were withdrawn from each side of the system at times 0, 15 min, 30 min, 1 h, 2 h, 4 h and 24 h. Two replicates were performed for twin bottles, dialysis inside and outside, and bioreactor. Three replicates were performed for the Transwell system. The sample volume was 1 mL for both the twin-bottles and the bioreactors. For Transwell and dialysis, 50 µL samples of each compartment were taken and diluted to 1 mL in water. All samples were centrifuged at 13,000 × g for five minutes to remove debris. The resulting supernatant was stored at -20 °C prior to HPLC analysis. Leakage of yeast cells was ruled out by microscopic examination of the pellets from the non-inoculated side.

For the fermentation experiments, MS300 was filled into both sides of each system in the volumes mentioned above. *S. cerevisiae* strain FX10 was inoculated in only one compartment at a final OD equivalent to 0.2 for the total volume of the system. The other compartment was not inoculated, and the absence of yeast contamination was monitored by OD measurements and microscopic examination throughout the experiment. Both compartments were sampled at 0, 7, 24, 33, 48, 55, 72, 96 and 168 h. Sample volumes were as described above. Experiments were run in three independent replicates. Transwell were used as provided and experiments were incubated at 25 °C without shaking. Dialysis systems were prepared for use under axenic conditions as follows. After preparing the membranes, the tubes and the membranes were filled with 70% ethanol for 20 min. Then, the 70% ethanol was substituted with 20% ethanol for 20 min. Both tubes and membranes were rinsed with sterile water for 5 min before filling with sterile synthetic must. The system was placed in an incubator at 25 °C with magnetic stirring. Finally, bioreactor systems were prepared by autoclaving the bioreactor vessels and headplates, while the CFF devices and connecting tubing were cleaned with NaOH 0.5 N, sterile water, ethanol 15% and sterile water. To avoid a dilution of the media, the membrane and tubing was flushed with sterile synthetic must. Fermentation tests were performed with temperature control at 25 °C, 1000 rpm stirring, and a nitrogen flow of 10 VVH (volumes of nitrogen per volume of media per hour in the headspace), to ensure that the process was anaerobic.

### Analysis of main fermentation-related metabolites

The concentration of glucose, fructose, glycerol, ethanol, and acetic acid was determined using a LC-2050C 3D liquid chromatograph (Shimadzu, Kyoto, Japan) equipped with a refraction index detector (RID 20A) on a 300 × 7.7 mm PL Hi-Plex H + (8 μm particle size) column (Agilent Technologies, Santa Clara, CA) and 4 × 3 mm ID Carbo-H guard (Phenomenex, Torrance, CA). The column was maintained at 50 °C and 1.5 mM H_2_SO_4_ was used as the mobile phase at a flow rate of 0.6 mL/min. Prior to injection in duplicate, the samples were filtered through 0.22 μm pore size nylon filters (Membrane Solutions, Auburn, WA).

### Volatile compound analysis

Samples for gas chromatography-mass spectrometry (GC–MS) analysis contained 1000 µl of the original sample and 20 µl internal standard in a final volume of 2000 µl completed with water, 1 g NaCl, in 20 mL flasks. Internal standard contained 1000 ppm each of 4-methyl 2-pentanol and heptanoic acid, and 100 ppm 1-nonanol, in water, prepared from a 100X solution in ethanol. Sample was preincubated for 10 min at 45 °C, followed by 30 min at 45 °C with 50/30 µm DBV/CAR/PDMS SPME fiber (Stableflex, SUPELCO, Bellefonte, PA). Fiber was desorbed for 5 min at 250 °C.

GC–MS was carried out in a Thermo TRACE GC Ultra apparatus coupled to a Thermo ISQ mass detector, equipped with a Thermo TriPlus autosampler. Gas chromatography was carried in a Thermo Scientific fused-silica capillary column TG-WAXMS A (30 m long; 0.25 mm OD; 0.25 µm film thickness). Chromatographic conditions were as follows: 5 min at 40 °C, 3 °C /min up to 200 °C, 15 °C /min up to 240 °C, 10 min at 240 °C. Helium was used as carrier gas at a flow rate of 1 mL/min, operating in split mode (ratio 30). Total analysis time was 71 min. Detection was performed with the mass spectrometer operating in the Full Scan mode (dwell time 500 ms), with 70 eV ionization energy, and source and quadrupole temperatures of 250 °C. Detection was stopped during the time interval for ethanol elution. Peak identification was made by comparison of ion spectra with NIST mass spectral library. For each compound, including internal standards, the sum of the areas of the peaks of selected characteristic ions was obtained^15^.

## Results

### Metabolite exchange kinetics using a model solution

**Fig. 2:**
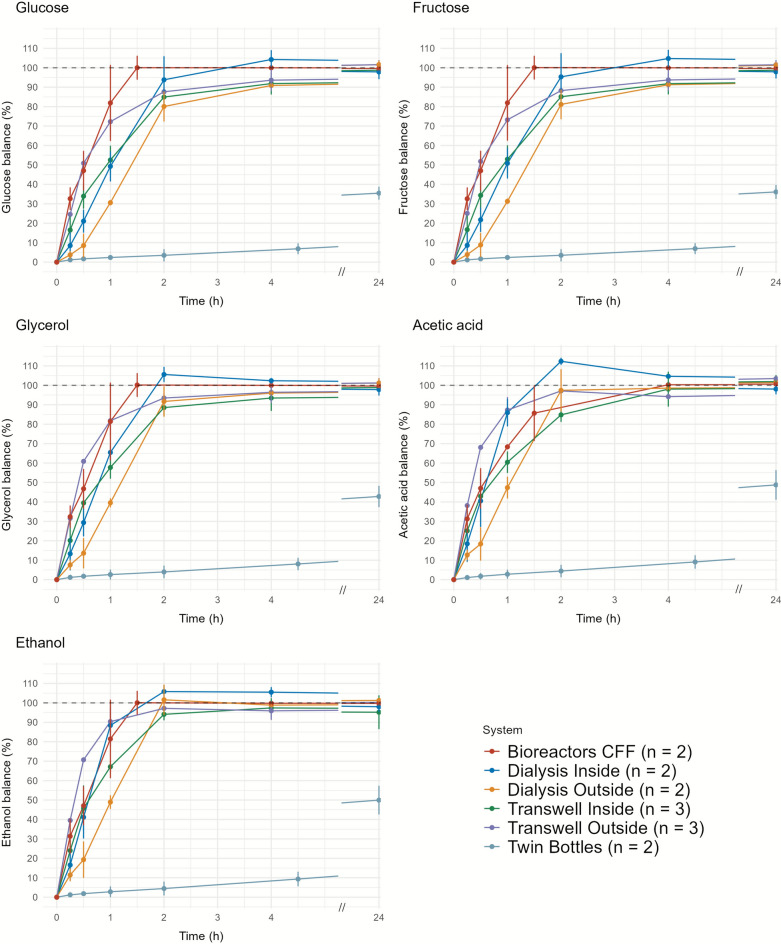
Metabolite transfer rate for the systems tested with water and the model solution. Y axis represents the percentage from equilibrium between compartments. The percentage is calculated as the ratio of the metabolite concentration between the receiving compartment and the source compartment. The values represent the mean of the experimental replicates (shown as ‘n’ in the graphical legend). Vertical bars represent standard deviation in the systems with three replicates and the individual values in the systems with two replicates.

The transfer rate of metabolites included in the model solution was relatively fast for most systems but for the twin-bottles (Fig. 2). For these systems glucose, fructose, ethanol, acetic acid, and glycerol were at or near equilibrium between 1.5 and 2.0 h in almost all of them. However, even after 24 h, the twin-bottles were still far from reaching equilibrium. The concentration in the receiving bottle was less than half that in the source bottle for any of the five metabolites analysed (Fig. 2). Accordingly, this system was discarded as a suitable model to study interactions in the target fermentation conditions.

There were also slight differences in the exchange rates of the other systems. For example, the fastest exchange rates for glucose, fructose and glycerol were found in the bioreactors, while the dialysis system showed the lowest exchange rates for all metabolites when the outside compartment was used as the source (Fig. 2). Indeed, the dialysis and Transwell systems show noticeable differences in the exchange kinetics depending on where the model solution is placed. Despite these differences, dialysis tubes, Transwell and bioreactors were all considered suitable for testing under fermentation conditions.

### Fermentation kinetics in selected double compartment systems

Fermentation experiments, with inoculation on only one side of the respective system, were carried out in bioreactors, dialysis tubes, and Transwell. For Transwell, both sides were alternately tested for inoculation, while dialysis tubes were assayed only for outer compartment inoculation.

**Fig. 3:**
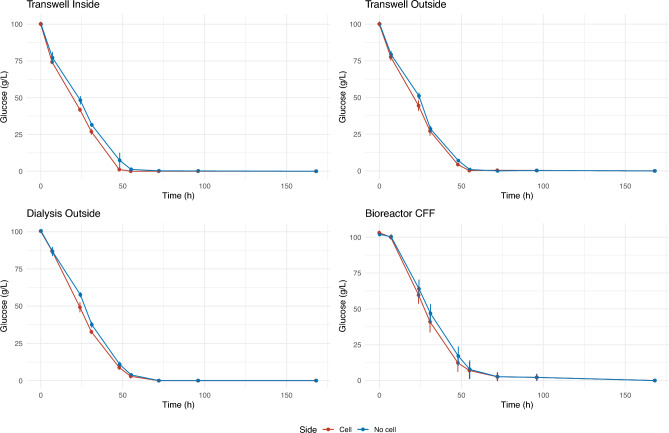
Glucose concentration through the fermentation process. Each panel represents one system. Vertical bars represent standard deviation between three replicates.

Fermentation kinetics was comparable in the four systems, according to glucose consumption profiles (Fig. 3), although bioreactors were slightly delayed compared to the other systems for the final steps, probably due to ethanol inhibition (see below). In all instances, the exchange of glucose between the cell-containing and non-inoculated compartments was quick. Only in the middle stages of fermentation (24 to 48 h) a slightly lower glucose concentration was observed on the inoculated side of each culture system, where sugar consumption takes place. As expected for the yeast strain used in these experiments, fructose consumption was somewhat slower than that of glucose. Again, a quick exchange of fructose was observed, with moderately faster consumption on the inoculated side in some cases (Figure S1).

**Fig. 4:**
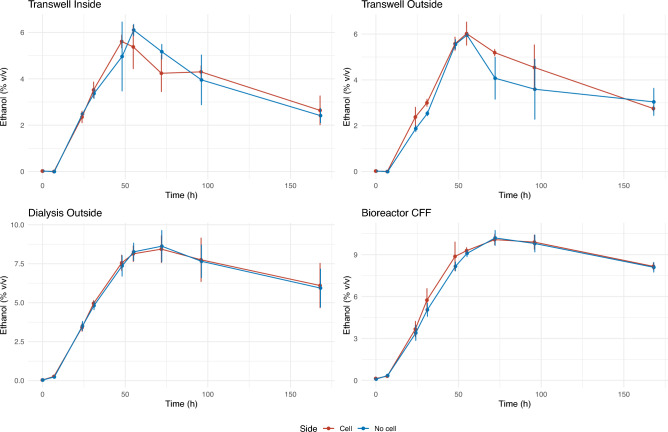
Ethanol concentration through the fermentation process. Each panel represents one system. Vertical bars represent standard deviation between three replicates.

The ethanol content was also well balanced between the compartments in the four experimental conditions throughout the fermentation experiments (Fig. 4). However, it differed greatly between the different systems. Transwell system reached a maximum ethanol content of around 6% (v/v), which falls far from the theoretical value for the amount of sugar consumed (11–11.5%). The dialysis system performed better, reaching almost 9% ethanol (v/v). Finally, despite stirring and nitrogen flow, ethanol in the bioreactors was better preserved than in the other two systems (Fig. 4), with maximum values close to 10% (v/v). This might have contributed to the longer time required for sugar depletion in this system. The differences in maximum ethanol levels appear to be due to evaporation, as they correlate with the surface/volume ratios in the different systems. Ethanol evaporation levels would also explain the data dispersion in Transwell and dialysis systems, which was not observed for sugars. The other two fermentation products analysed in this work, glycerol and acetic acid, were equally well balanced for all systems along the fermentation (Figure S1). No losses were observed after sugar depletion, and even an increase in acetic acid concentration was observed in the Transwell experiments, which were more exposed to oxygen than the other two systems.

The final sample of dialysis and bioreactor systems was also analysed for the distribution of volatile compounds. Due to the volume limitation of the Transwell system, volatile compounds were not quantified for this system. Thirty-three compounds were quantified, 14 esters, 10 alcohols, 6 acids, and 2 aldehydes. To contrast the transfer efficiency between systems, the concentration of each compound was compared between the two compartments (Table S1).

The groups of alcohols (1-propanol, 1-butanol, 2-methyl propanol, 2-methyl butanol, 3-methyl butanol, 1-hexanol, 2-ethyl hexanol, 2-phenyl ethanol, benzyl alcohol, methionol) and of aldehydes (acetaldehyde, benzaldehyde) showed a homogeneous distribution between compartments, both in bioreactors and in the dialysis system.

The group of acids can be divided into two categories. For iC4, iC5, and C6 acids there are no differences in concentration between compartments with and without cells in any of the systems tested, as described for alcohols. In contrast, for C8, C9, and C10 acids, dialysis samples showed higher concentration in the inner compartment, where the cells are located.

In the esters group, diverse behaviours were found. For acetate esters, as for alcohols, the relative concentration is close to 100% in both systems, while for most ethyl esters, the bioreactor shows a better balance than the dialysis system.

## Discussion

Dual-compartment devices are a popular first approach for studying the impact of cell-to-cell contact on microbial interactions. However, the heterogeneity of conditions and the difficulty of replicating the same system make reproducibility and comparability challenging when developing a new experimental design. Effective exchange of solutes is typically taken for granted or confirmed by comparing the concentration of selected metabolites in compartments inoculated with different microbial strains. However, comparing the concentration of the main fermentation metabolites when both sides of the system are inoculated could lead to erroneous conclusions about the actual exchange rates since both compartments can be fermenting with similar kinetics. This may result in cell-to-cell contact being deemed essential for a given interaction, when it could actually depend on small molecules or structures that cannot cross the separation barrier effectively. For this reason, we have designed our experiments by inoculating one single compartment in the systems.

Diffusion based systems similar to the twin-bottle tested in this work have been used by several authors to study interactions during fermentation of synthetic grape must^16–18^. Our results show that, with different solutions (model solutions and water), the time to equilibrate both compartments exceeds 24 h. This precludes any conclusion on the impact of cell-to-cell contact using this system. Additionally, this type of system relies on custom-made devices, which may be homemade or produced by local companies. This creates an additional barrier to inter-laboratory reproducibility. Also, depending on the device, the tightness of the compartments must be regularly checked to prevent the passage of medium around the membrane. In some cases, diffusion-based systems have been combined with active exchange^10^. In principle, the behaviour of these latter systems should be closer to the bioreactor system used in this work.

The Transwell system is broadly used for cell migration and invasion assays^19^ but, as far as we know, this system has not been tested with grape must or other types of food fermentation. The exchange rate seems to be good enough for short culture times, and the different Transwell formats available (from 1 to 96 wells) provide versatility to this system. However, the relatively large surface-to-volume ratio compared to other systems leads to a rapid loss of volatile compounds, such as ethanol. This introduces interferences, particularly in fermentation experiments. The available range of pore sizes (0.4–8.0 µm) allows for the investigation of cell-to-cell contact. However, it does not permit the evaluation of the impact of smaller structures or molecules. Finally, the small working volumes of Transwell limit the sampling capacity for fermentation studies, which can take more than one week to complete.

The dialysis system has been widely used to study interactions between wine yeasts^8,20–24^. One drawback of this setup is the difficulty of ensuring axenic conditions through the whole fermentation, and it is difficult to accurately replicate the system between laboratories. Using ready-to-use dialysis systems can help solve some of these problems. There is a range of cut-off sizes for dialysis tubing, from 0.1 to 1000 kDa, allowing to target different molecules and protein sizes. In this work, using a cut-off size of 1000 kDa, the transfer rate for most metabolites was good, although there are limitations for some volatile compounds. This system performed better than Transwell concerning ethanol evaporation.

Given that the GC–MS samples were analyzed at the final time point (168 h), the imbalance observed for some volatile compounds may suggest an interaction with the membrane formulation. The potential interaction between the metabolites of interest and the membrane should be considered during the experimental design process.

Out of the systems that were tested in this study, the active exchange in bioreactors, which consists of two vessels connected by two hollow fiber CFF systems (Fig. 1), was the best on several criteria, such as the metabolite exchange rate, ethanol evaporation, and a working volume that is suitable for relatively large sample sizes or sampling frequencies in long experiments. The use of hollow fiber filters has been proposed in the year 2000 and used by several authors^7,25–32^. A similar alternative is the use of cross-flow ceramic membranes^33^. The wide range of cut-off sizes available for this system allows, in principle, for the differentiation of the effect of actual cell-to-cell contact. For example, a 0.65 µm cut-off can be used, as is the case in this work. Alternatively, superstructures such as extracellular vesicles can be targeted, using a 100 kDa cut-off. Finally, the exchange can be limited down to 3 kDa to test hypotheses about smaller molecules. Such a broad range of cut-off sizes is not available for any of the other systems. However, it is the most expensive system because it requires relatively costly equipment and supplies. This could pose an entry barrier for some laboratories. Since the cross-flow filtration can generate shear stress on yeast cells, it is also important to design appropriate controls for each experimental setup. Depending on the type of media and strains utilised, the system may be susceptible to membrane fouling. To prevent degradation of the membrane’s performance, this effect should be considered and act in consequence, by, for example, back flushing the membrane. The ability to form biofilms by some yeast strains should be also taken into account, as it can affect the capacity of exchange of the filter in long experiments or generate subpopulations with different metabolic features.

In summary, these three systems allow an effective exchange between compartments during fermentation and present their own advantages and limitations depending on the type of the study designed.

## Conclusions

The study of the role of cell-to-cell contact in microbial interactions is a challenging field, especially in non-standard culture conditions, such as research on wine fermentation. The ability to physically separate cells by keeping them in the same medium maintaining a homogenous composition in real time – even with high metabolic activity – is crucial to advance the study of interactions. The results shown in this article highlight the need to carefully study whether the system chosen for a given experimental design is adequate. Each system has its own capabilities and limitations. To avoid erroneously concluding that cell-to-cell contact (or other molecules or structures, such as extracellular vesicles) is involved in interspecies microbial interactions, it is recommended to verify that the exchange rates of the chosen compartmentalization system are suitable for the experiment to be performed.

## Supplementary Information

Supplementary Information 1.

Supplementary Information 2.

## Data Availability

All the data used in this work are included in the article or the supplementary material.
