# Supplementary material for: Evaluation of compartmentalization systems to study microbial interactions
Source: Sci Rep. 2025 Dec 18;16:2713. doi: 10.1038/s41598-025-32560-3 (PMC12824151; doi:10.1038/s41598-025-32560-3)
Supplement: Supplementary file 1 — Supplementary Information 1. [file 41598_2025_32560_MOESM1_ESM.pdf]

Figure S1. Fructose, glycerol and acetic acid concentration through the fermentation. Each panel represents one system. Vertical bars represent standard deviation.

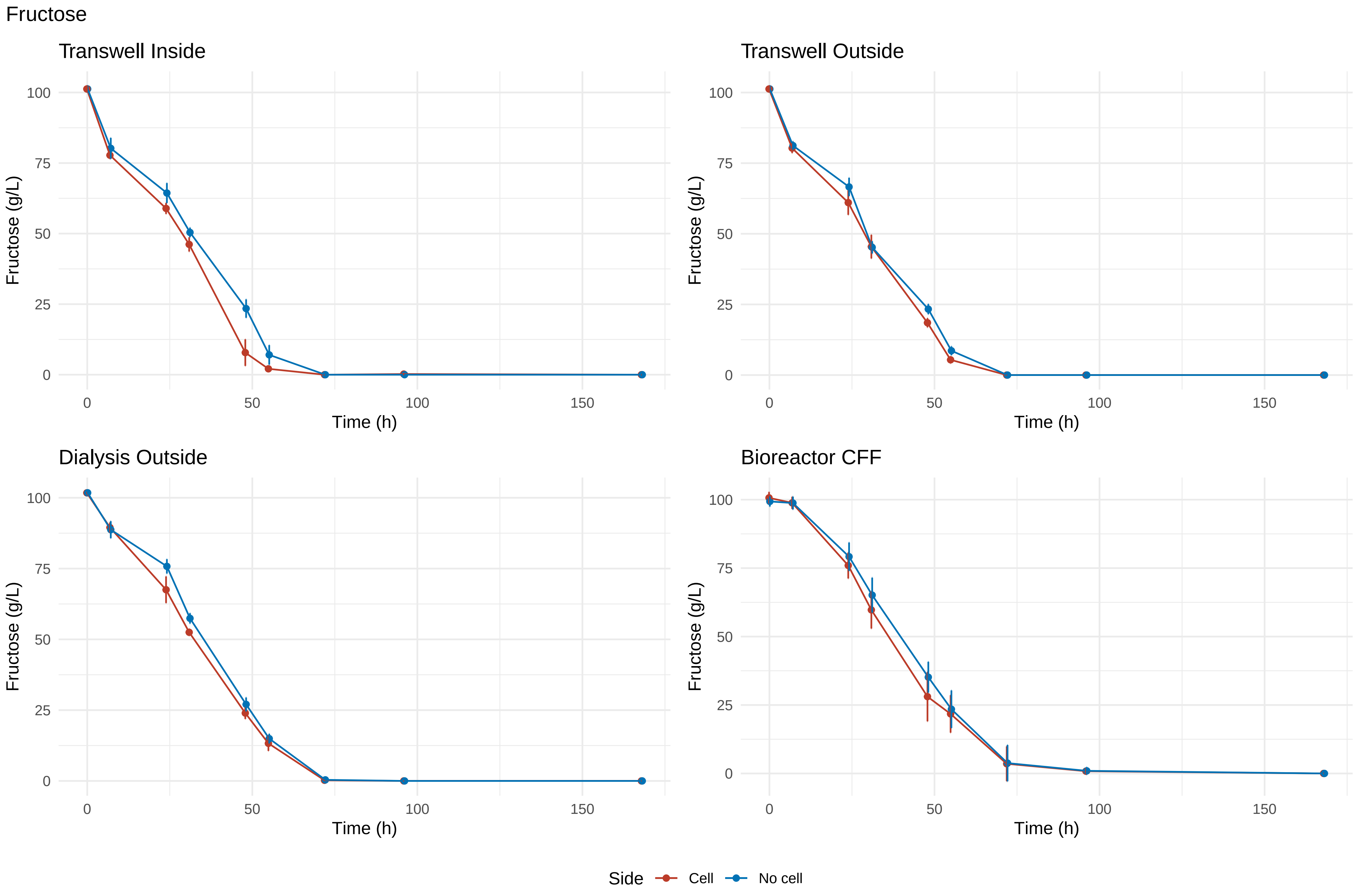
Fructose
Transwell Inside
Transwell Outside
Dialysis Outside
Bioreactor CFF
Fructose (g/L)
Time (h)
0
25
50
75
100
150
Side
Cell
No cell

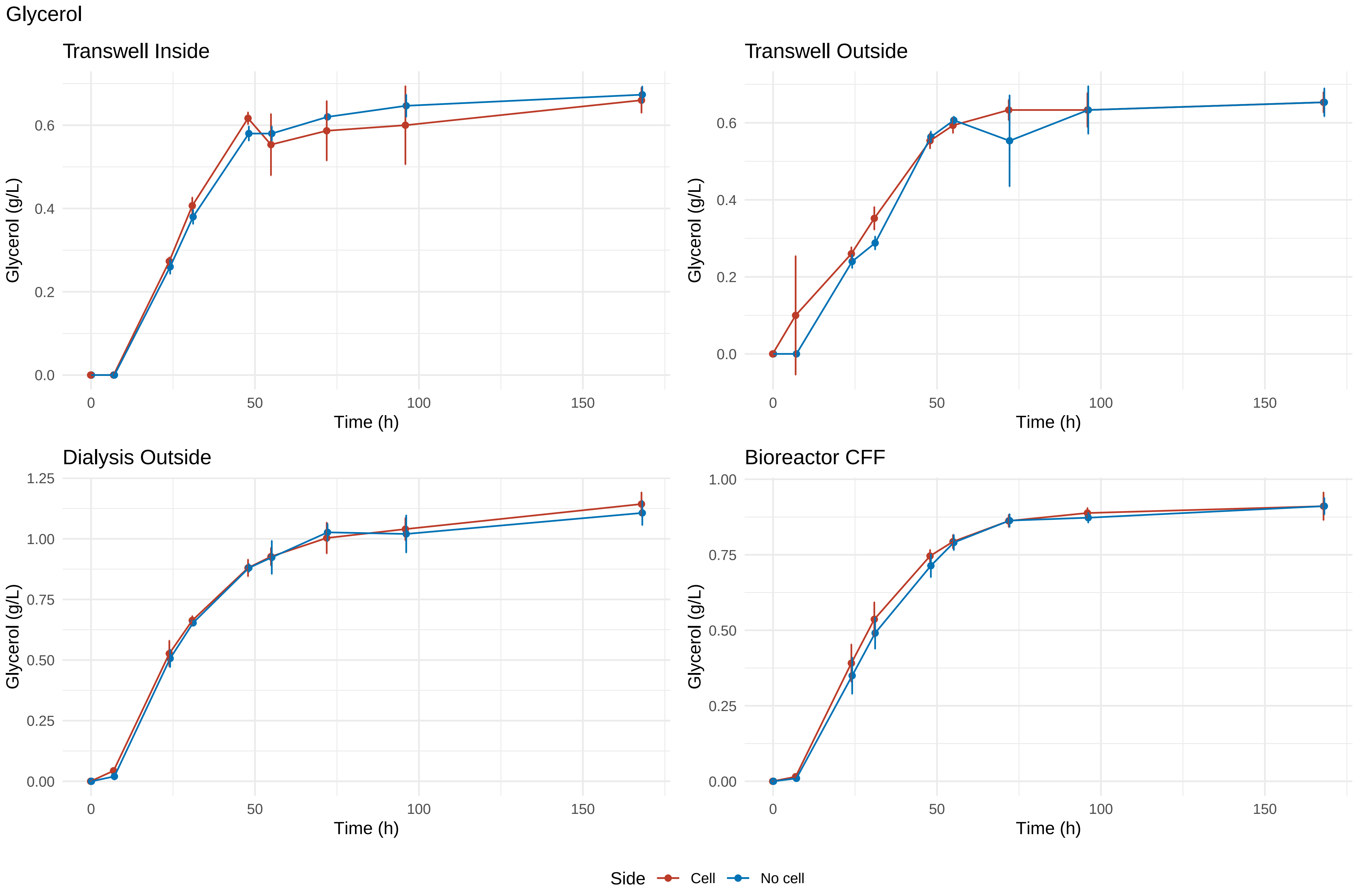
Glycerol
Transwell Inside
Transwell Outside
Dialysis Outside
Bioreactor CFF
Glycerol (g/L)
Time (h)
0.0
0.2
0.4
0.6
0.00
0.25
0.50
0.75
1.00
1.25
0
50
100
150
Side
Cell
No cell

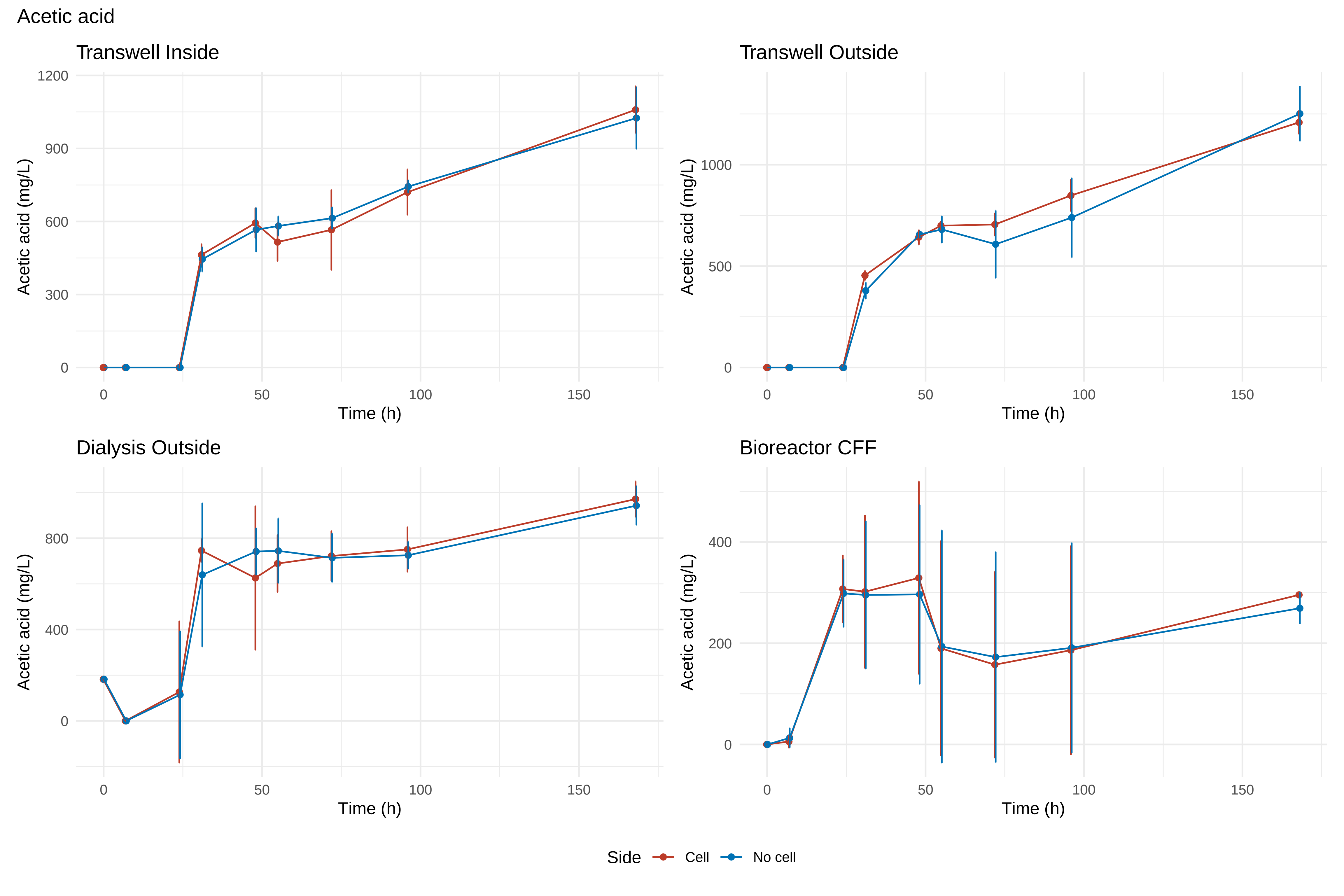
Acetic acid
Transwell Inside
Transwell Outside
Dialysis Outside
Bioreactor CFF
Acetic acid (mg/L)
Time (h)
0
50
100
150
0
300
600
900
1200
500
1000
-400
400
800
200
400
Side
Cell
No cell
